# *Escherichia coli* Infection Sepsis: An Analysis of Specifically Expressed Genes and Clinical Indicators

**DOI:** 10.3390/diagnostics13233542

**Published:** 2023-11-27

**Authors:** Qingyi Shao, Danlei Chen, Simiao Chen, Xuanwen Ru, Qing Ye

**Affiliations:** The Children’s Hospital, Zhejiang University School of Medicine, National Clinical Research Center for Child Health, National Children’s Regional Medical Center, Hangzhou 310052, China

**Keywords:** sepsis, *E. coli*, immune cell infiltration, WGCNA, inflammation

## Abstract

Since *E. coli* is the most prevalent sepsis bacterium, studying its pathogenic molecular pathways may help with its early diagnosis and individualized treatment. However, few studies have investigated the molecular characterization of *E. coli* infection only. We extracted *E. coli* infection-specific genes and indicators from published data and clinical laboratory results in this study. GSE65088 showed 277, 377, and 408 DEGs for *E. coli* and other bacteria, *E. coli* and healthy groups, and other bacteria and healthy groups, respectively. DEGs, the MEgreen module with the highest relevance in WGCNA, and the first three MCODE subnetworks were used to find *E. coli* infection-specific hub genes. HSPA1B and TNF were verified in GSE6269 with ROC-AUCs of 0.7038 and 0.7116, respectively. CIBERSORT showed increased B-cell naive and T-cell CD4 naive infiltration in *E. coli* infectious sepsis. Patients infected with *E. coli* were younger than those infected with other pathogens. Compared to the other bacterially infectious sepsis patients, the *E. coli* patients had low globulin, prealbumin, creatine kinase, and high bilirubin levels. The clinically significant difference indicator IL-2, in combination with hub genes, better differentiated the healthy and *E. coli* groups, with an ROC-AUC of 0.8793. The study suggested that HSPA1B and TNF may be *E.-coli*-infection-specific genes, which may help explain the molecular mechanism of infectious sepsis.

## 1. Introduction

Despite significant progress in the study of how pathogens cause disease and the development of treatments to fight infections, infections continue to be the main cause of death today [1,2]. Sepsis is a life-threatening organ dysfunction, mainly due to the dysregulated host response to infection, and it is an important global health problem [3,4].

Molecular patterns associated with pathogens can interact with particular pattern recognition receptors that are present in immune cells. Therefore, the blood immune cells of infected patients may have pathogen-specific transcriptional signatures [5]. In sepsis, the most prevalent bacteria that can cause serious clinical consequences are *Staphylococcus aureus* (*S. aureus*) and *E. coli* [6,7]. *E. coli* infection most commonly causes intestinal diseases, such as watery or bloody diarrhea, but few studies have solely positioned it in systemic infections such as sepsis [8,9]. The colonization and infection of extraintestinal locations via pathogenic *E. coli* are the leading causes of sepsis in hospitals and communities [10]. For instance, *E. coli* was the pathogen responsible for the majority of cases in newborns with preterm sepsis among ICU patients in Saudi Arabia. [11]. *E. coli* is also the most common cause of bacteremia in England, with an incidence rate of 50.7 cases/100,000 people [12]. *E. coli* sepsis is a systemic infectious disease caused by *E. coli*. Severe fever, chills, tachycardia, hypotension, and multiple-organ dysfunction syndrome are typical clinical symptoms. In addition, clotting problems and the inflammatory response that follows an infection can cause symptoms, including hemorrhage and rashes [13].

Although genome-wide investigations have revealed possible host genes for sepsis development, only a few studies have characterized the various pathophysiological mechanisms of infectious illnesses caused by Gram-positive and Gram-negative bacteria [14,15]. In addition, studies that have analyzed only *E. coli* infection characteristics are even scarcer. However, for patients with sepsis, initial diagnosis and appropriate medical care are essential to improve clinical outcomes [16]. Understanding the potential molecular mechanisms of *E. coli* infectious sepsis is critical for the identification of early diagnostic biomarkers, the development of individualized treatment strategies, and the search for effective drugs.

Some studies have analyzed the difference in innate immune activation in whole-blood-cell gene expression infected by bacteria (*S. aureus* or *E. coli*) and mock-treated blood. Thirty-eight biomarker genes, including IL6, SOCS3, and IRG1, were discovered in one study and were later related to sepsis in subsequent investigations [17]. In addition, another study analyzed the gene expression characteristics of the peripheral blood leukocytes of patients infected with different pathogens and identified 35 genes that could be used to differentiate different pathogens. These potential biomarkers were also used in the study of molecular mechanisms and therapeutic objectives [5].

In the present study, we also reviewed the data of a total of 109 septic patients with confirmed pathogens and divided them into an *E. coli* infection group, an other Gram-negative bacteria infection group, and a Gram-positive bacteria infection group. To investigate and contrast the clinical indicator traits of patients who were infected with *E. coli*, clinical indicator data from patients were gathered. At the same time, we used a variety of bioinformatics methods to analyze the signature genes of *E. coli* infection, and an outside dataset was used to confirm them. To provide physicians with novel ideas about diagnosis and treatment, we also assessed the enriched signaling pathways of these genes and their functions in immune cell infiltration.

## 2. Materials and Methods

### 2.1. Clinical Data: Study Cohort and Information Collection

Criteria for inclusion and exclusion: (1) Patients with positive blood cultures and with a clear pathogen of *E. coli* or other bacteria were included; (2) they met the diagnostic criteria of sepsis 3.0 [18]. (3) Patients with tumors or hematological diseases, (4) patients with autoimmune diseases, and (5) patients with chronic diseases requiring immunomodulatory therapy were excluded. The age, sex, and basic diagnostic information of the included population were collected. In addition, data on common clinical laboratory indicators, such as biochemistry, routine blood tests, C-reactive protein (CRP), procalcitonin (PCT), and cytokines, were obtained.

This retrospective cohort study was conducted at the Children’s Hospital affiliated with Zhejiang University (2023-IRB-0107-P-01). The hospital’s Medical Ethics Committee gave its approval. The 1975 Declaration of Helsinki and the ethical guidelines of the responsible committee were followed when conducting this investigation. All subjects or their legal guardians gave their written, fully informed consent.

### 2.2. Data Source

**Figure 1:**
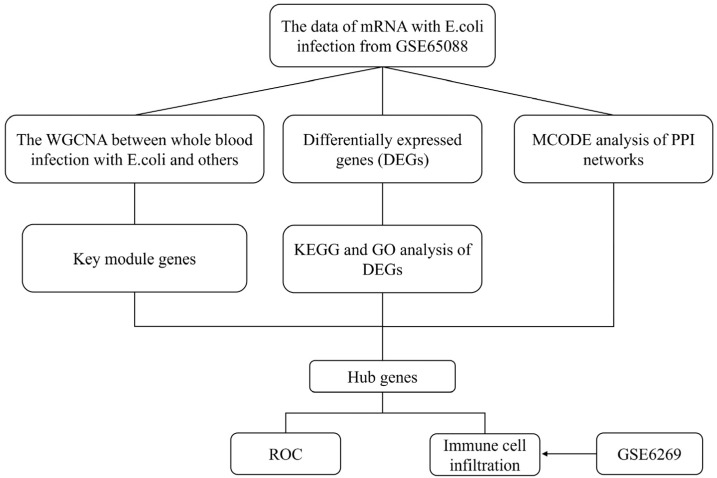
A flow chart illustrating the research findings of this study.

Two datasets for this study, GSE65088 and GSE6269, were obtained via a Gene Expression Omnibus (GEO) download. GSE65088 was used as a training set, in which 10 cases of *E. coli* were cocultured with peripheral blood cells, 20 cases of other bacteria were cocultured with peripheral blood cells, and there were 15 cases in the peripheral blood model control group. As a validation set, GSE6269 includes 29 cases of *E. coli* sepsis, 44 cases of other bacterial sepsis, and 6 cases of healthy controls. The flow chart details all data analysis processes (Figure 1).

### 2.3. DEG Identification and Functional Enrichment Analysis

The LIMMA software package of R software (version 4.1.0) was used to evaluate the differentially expressed genes (DEGs) between the *E. coli* infection group and the control group, the *E. coli* infection group and the other bacterial groups, and the other bacterial infection and the control group. The criteria were |fold change (FC)| > 1 and *p* < 0.05. The clusterProfiler package in R was used for functional enrichment analyses of DEGs based on gene ontology (GO) and the Kyoto Encyclopedia of Genes and Genomes (KEGG). The biological functions and signaling pathways involved in *E. coli* infection should be further studied.

### 2.4. PPI Network and MCODE Analysis

The protein–protein interaction (PPI) network of DEGs infected with *E. coli* was predicted via the Search Tool for the Retrieval of Interacting Genes/Proteins (STRING). Subsequently, a molecular interaction network was created and visualized using the Cytoscape (version 3.8.0) program. Target proteins are shown as nodes in the PPI network, while edges indicate potential or observed interactions between proteins. In addition, to cluster the PPI–protein interaction network to form multiple subnetworks, the molecular complex detection (MCDOE) plug-in was utilized, and the potential hub genes in the subnetworks were analyzed [19].

### 2.5. Weighted Gene Coexpression Network Analysis (WGCNA)

Based on the scale-free topology criterion, the coexpression network in the GSE65088 cohort was constructed using weighted gene coexpression network analysis (WGCNA). Employing the block module function, a scale-free network was developed, and gene coexpression modules were located through module partition analyses. Following the application of the dynamic tree-cutting technique, each module was defined via gene branching, and for the purpose of visualization, various colors were allocated to each module. Applying the dynamic tree-cutting method, we identified the coexpressed gene modules as having an acceptable module size of 50. Then, we assessed the relationship between the gene modules and *E. coli* infection using gene significance (GS) and module membership (MM), ultimately identifying the key modules [20,21].

### 2.6. Infiltration of Immune Cells in E. coli Sepsis

CIBERSORT (http://www.cibersort.stanford.edu/ (accessed on 11 July 2023)) is a technique that performs deconvolution on a gene expression matrix of 22 human immune cell subgroups utilizing linear regression based on the support vector regression principle. We used this method to assess immune cell infiltration in *E. coli* infectious disease [22,23]. The CIBERSORT program succeeded in deducing the corresponding proportions of 22 infiltrating immune cells, and the entirety of all predicted immune cell subtype scores in every sample was the same as 1. Additionally, a Spearman correlation study was conducted, comparing the levels of gene expression and the cellular composition of the immune system [24,25].

### 2.7. Statistical Analysis

Statistical analysis was carried out with the assistance of GraphPad Prism 8.0, SPSS 26.0, and R Program 4.0. An independent Student’s t-test was carried out to determine the statistical significance of variables with a normally distributed distribution for the contrast of two separate sets of continuous data. The Mann–Whitney U-test was applied to assess the distinction among nonnormally distributed data. The statistically significant effect of two separate sets of categorical variables was analyzed using either the chi-square test or Fisher’s exact test, depending on which method was preferred. The two-tailed distribution was implemented for all statistical *p*-values, and a value of *p* less than 0.05 was regarded as statistically significant.

## 3. Results

### 3.1. DEG Identification and Functional Enrichment

**Figure 2:**
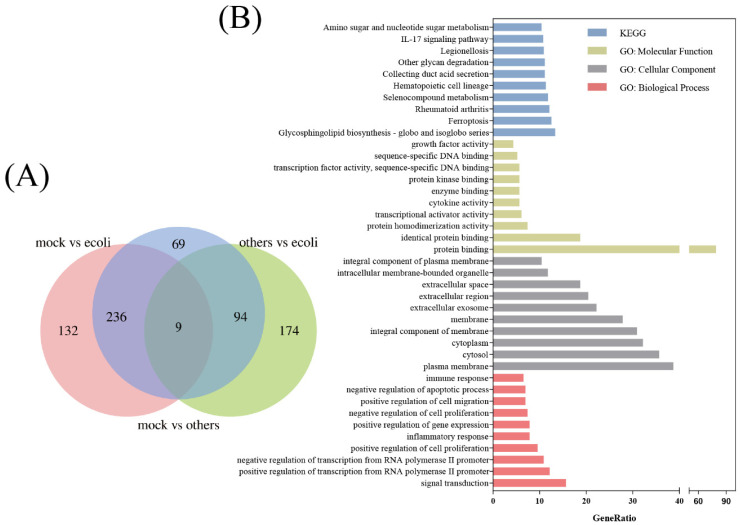
The determination of DEGs and their subsequent functional enrichment. (**A**) The Venn diagram shows the DEGs. The mock vs. ecoli group comprised the genes whose expression levels differed between the *E. coli* infection group and the healthy model, the mock vs. others group comprised the genes whose expression levels varied between the other bacterial infection groups and the healthy model, and the others vs. ecoli group comprised the differentially expressed genes between other bacteria and *E. coli*. (**B**) Top 10 functional enrichment items in the KEGG, MF, CC, and BP analyses of DEGs.

The flow chart details all data analysis processes (Figure 1). The GSE65088 and GSE6269 datasets related to *E. coli* infection were downloaded from the GEO database. Overall, 10 peripheral blood samples infected with *E. coli*, 15 mock blood samples, and 20 peripheral blood samples infected with other bacteria were included in GSE65088. Through screening with the LIMMA software package in R software, 377 (mock vs. *E. coli*), 409 (mock vs. others), and 277 (*E. coli* vs. others) differentially expressed genes were identified, with 9 genes being common DEGs (Figure 2A). Furthermore, functional enrichment analysis using GO and pathway analysis using KEGG were carried out across the DEGs from *E. coli* vs. the others, suggesting that signal transduction, plasma membrane, cytosol, cytoplasm, the integral component of the membrane, protein binding, and glycosphingolipid biosynthesis—globo and isoglobo series—may play significant parts in *E. coli* infection (Figure 2B).

### 3.2. MCODE Analysis

**Figure 3:**
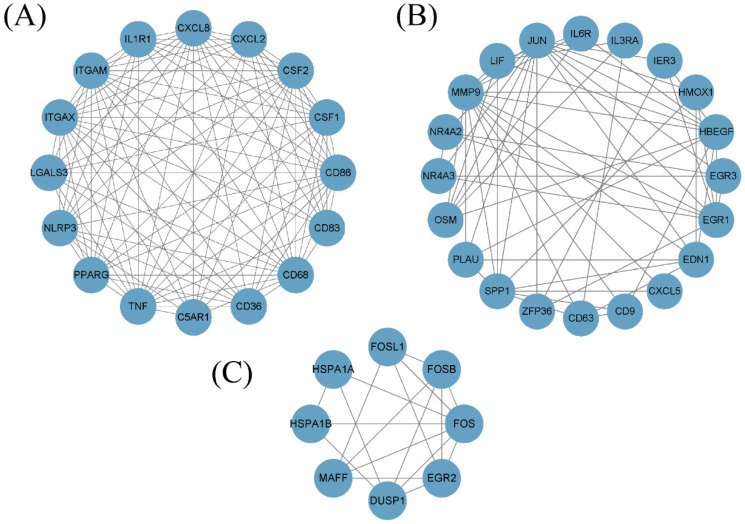
MCODE analysis built around data collected via the PPI network. (**A**) MCODE1. (**B**) MCODE2. (**C**) MCODE3.

By analyzing the protein–protein interaction network (PPI) represented by 277 DEGs using STRING, we discovered that the majority of increased proteins established an extensive network of interactions. To further analyze the subnetwork of differentially expressed genes, the molecular complex detection (MCODE) plug-in (version 1.5.1) was used within the Cytoscape software (version 3.8.0). Next, we proceeded to choose the three most prominent protein complex modules, as depicted in Figure 3A–C. The key or pivot modules in this context are MCODE1, MCODE2, and MCODE3. MCODE1 contained CXCL8, CXCL2, CSF2, CSF1, CD86, CD83, CD68, CD36, C5AR1, TNF, and IL1R1. MCODE2 contained IL6R, IL3RA, IER3, HMOX1, HBEGF, EGR3, EGR1, END1, CXCL5, CD9, and CD63. MCODE3 included FOSL1, HSPA1B, and HSPA1A.

### 3.3. Weighted Gene Coexpression Network Analysis (WGCNA) of E. coli Infection and Other Bacterial Infections

**Figure 4:**
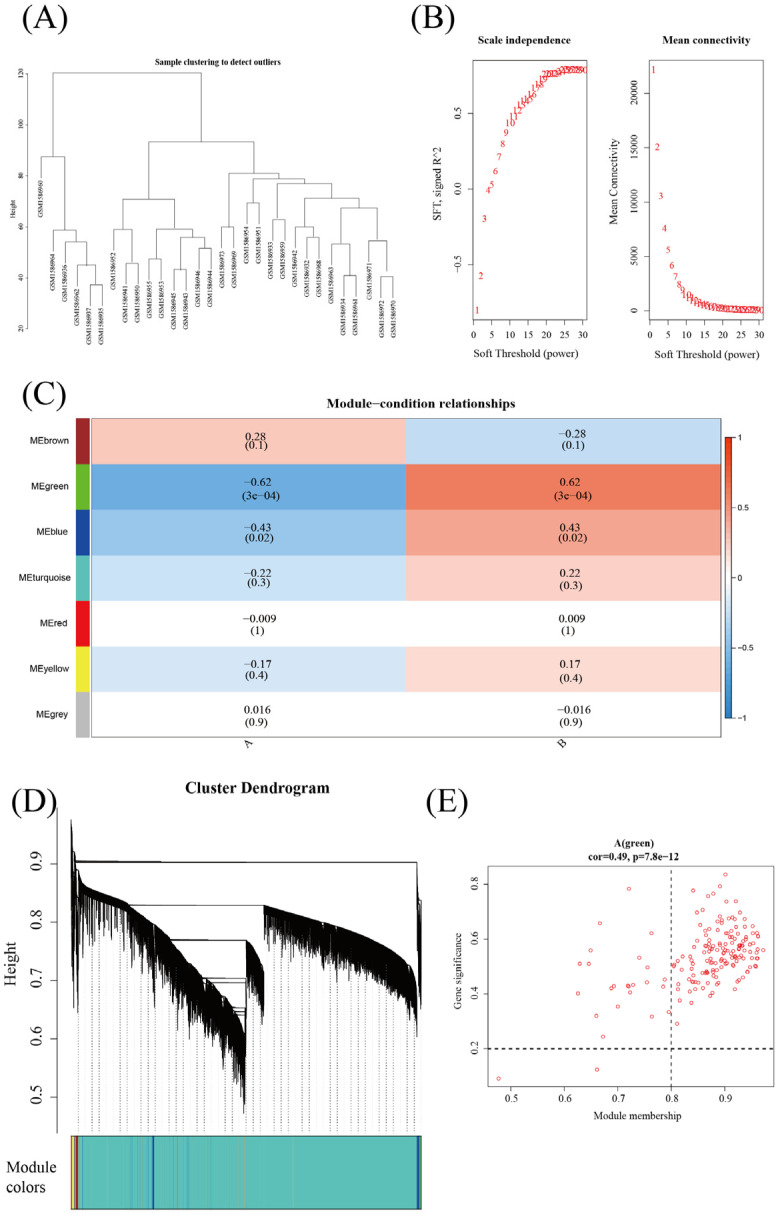
Module construction and assessment of WGCNA. (**A**) Dendrogram for specimen clustering utilizing Euclidean distances. (**B**) Analysis of the network architecture with a variety of different soft thresholds. (**C**) Module–trait association. A module is represented by each row, while a group is represented by each column. (**D**) Using module color assignment and topology connect, a clustered tree comprising various genes was constructed. (**E**) The relevance of members in the MEgreen module and sepsis.

Cluster analysis was performed on the gene expression profiles of the *E. coli* infection group and other bacterial infection groups (Figure 4A). To avoid the network from expanding, we selected a soft threshold (b = 6) (Figure 4B). The expression matrix was, next, transformed into an adjacency matrix, and finally, it was transformed into a topographical matrix. Genes were subjected to cluster analysis using the average linkage phylogenetic clustering method. The minimal number of genes needed for each gene network module was established as fifty with reference to the hybrid dynamic pruning tree as the benchmark. Seven modules were identified with these criteria: height = 0.10 and depth split = 2. The highest correlation with sepsis was MEgreen, r = −0.62 and *p* = 3 × 10^−4^ (Figure 4C). The clustering tree diagram is shown in Figure 4D. The genes associated with the MEgreen module and *E. coli* infection were significant with COR = 0.49 and *p* = 7.8 × 10^−12^ (Figure 4E).

### 3.4. Potential E. coli Hub Genes

**Figure 5:**
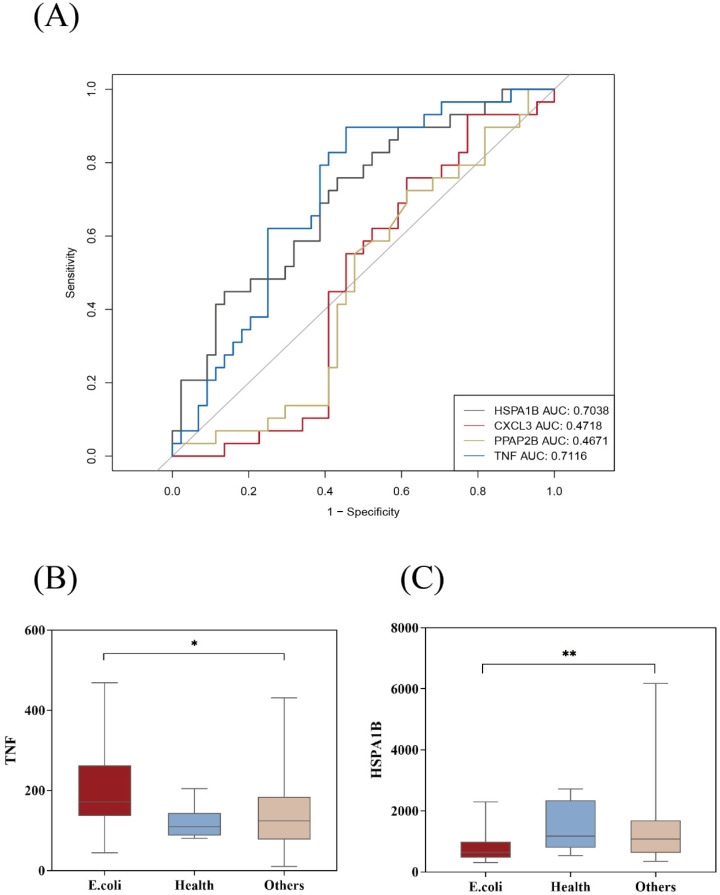
The performance of the hub genes in GSE6269. (**A**) ROC curve of the hub genes. (**B**,**C**) The expression of hub genes between *E. coli* and other cohorts. The Others group was the other bacterial infections group. *, *p* < 0.05; **, *p* < 0.01.

Table 1 lists the nine genes screened in Figure 2. The four genes in the WGCNA MEgreen module were HSPA1B, CXCL3, PPAP2B, and TNF (Table 1). HSPA1B and TNF were found in the first three subnetworks of MCODE. We investigated the identification of the above genes in the GSE6269 dataset for the occurrence and development of *E. coli* infection via ROC curve analysis. The findings revealed the following AUCs for the four genes: HSPA1B AUC: 0.7038, CXCL3 AUC: 0.4718, PPAP2B AUC: 0.4671, and TNF AUC: 0.7116 (Figure 5A). In the validation set, compared to the other bacterial infection groups, we discovered that the *E. coli* infection group had a greater TNF level (*p* < 0.05), while the HSPA1B level was diminished in comparison to the other bacterial groups (*p* < 0.01) (Figure 5B–C).

### 3.5. Immune Cell Infiltration

**Figure 6:**
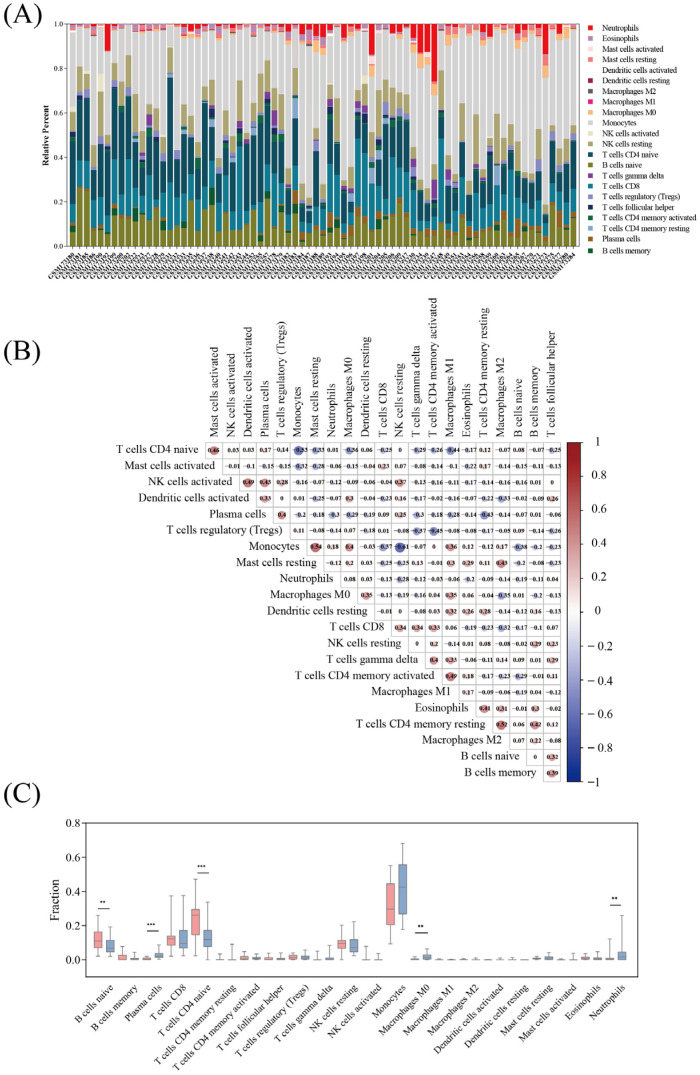
Assessment of the invasion of immune cells and correlation studies. (**A**) Bar plot showing the percentages of 22 distinct immune cells in specimens from patients with sepsis. (**B**) The correlation heatmap of 22 various forms of immune cell infiltration. Blue indicates a positive correlation, and red indicates an adverse association. A greater correlation is indicated by a deeper color. (**C**) The expression of 22 various forms of immune cells in the *E. coli* infection group and the other bacterial infection groups. The other bacterial group is shown in blue, while the *E. coli* group is shown in red. **, *p* < 0.01; ***, *p* < 0.001.

The proportions of various immune cell types in the peripheral blood of the *E. coli*-infected and other bacterial infection groups were assessed using CiberSort. GEO transcriptional microarray information was used to analyze the infiltration rate of immune cells. Among 73 specimens, there were 44 cases in the other bacterial infection group and 29 cases in the *E. coli* infection group, which met the CiberSort criterion (*p*-value < 0.05). The ratio of immune cells was significantly different within the groups (Figure 6A). We performed a correlation investigation of the immune cells in the *E. coli* infection group and discovered numerous combinations of immune cells with positive and negative correlations (Figure 6B), with the correlation’s degree indicated by the score. The synergy between monocytes and resting mast cells was the strongest, and the competition between monocytes and resting NK cells was the strongest. In contrast to the other categories of bacterial infections, the *E. coli* infection group had significantly increased naive B cells and naive CD4 T cells and significantly reduced plasma cells and neutrophils (Figure 6C).

### 3.6. Relationship between Immunological Cells and Hub Genes

**Figure 7:**
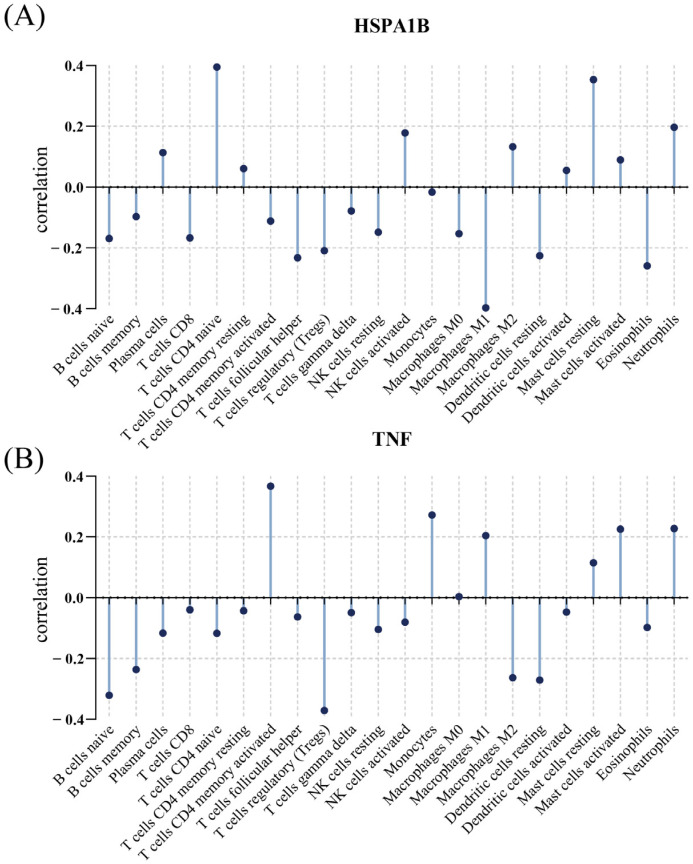
Correlation between immune cells and two genes. (**A**) Correlation between immune cells and HSPA1B. (**B**) Correlation between immune cells and TNF.

In the present research, we examined the relationship in the *E. coli* infection dataset between the hub gene and immune cell infiltration, thus further exploring the potential molecular mechanisms by which the hub gene affects *E. coli* infection. The two genes were both closely associated with immune cells, according to a correlation investigation with immune cells. Furthermore, Figure 7A–B’s data demonstrate an adverse association between TNF and regulatory T cells (Tregs) and an upward association with activated memory CD4 T cells (*p*-value < 0.05). HSPA1B demonstrated an adverse relationship with M1 macrophages and a positive relationship with naive CD4 T cells (*p*-value < 0.05).

### 3.7. Clinical Features of E. coli Infection

We enumerated the specific values of the indicators for which the differences were significant (Table 2). The age of patients infected with *E. coli* was notably lower than that of patients affected by other Gram-positive bacteria. For biochemical indicators, the peripheral albumin and globulin contents of patients infected with *E. coli* were significantly lower than those of patients with other bacterial infections, while total bilirubin and indirect bilirubin were significantly higher than those of patients with other bacterial infections. In addition, the levels of adenosine deaminase, prealbumin, and CK in the *E. coli* infection group were significantly lower than those in the Gram-positive coccus infection group (*p* < 0.05). For blood cell counts, the proportion of neutrophils in the *E. coli* infection group was significantly lower than that in the Gram-positive bacteria infection group, while the proportion of monocytes was significantly higher than that in the Gram-positive bacteria infection group (*p* < 0.05). Interleukin 2 (IL-2) in the *E. coli* infection group was higher than that in the Gram-positive bacteria infection group (*p* = 0.023). The TNF level of the clinical *E. coli* infection group was also slightly lower than that of the Gram-positive bacterial infection group, but the difference was not significant (*p* = 0.332).

### 3.8. Combined Analysis with Clinical Practice

**Figure 8:**
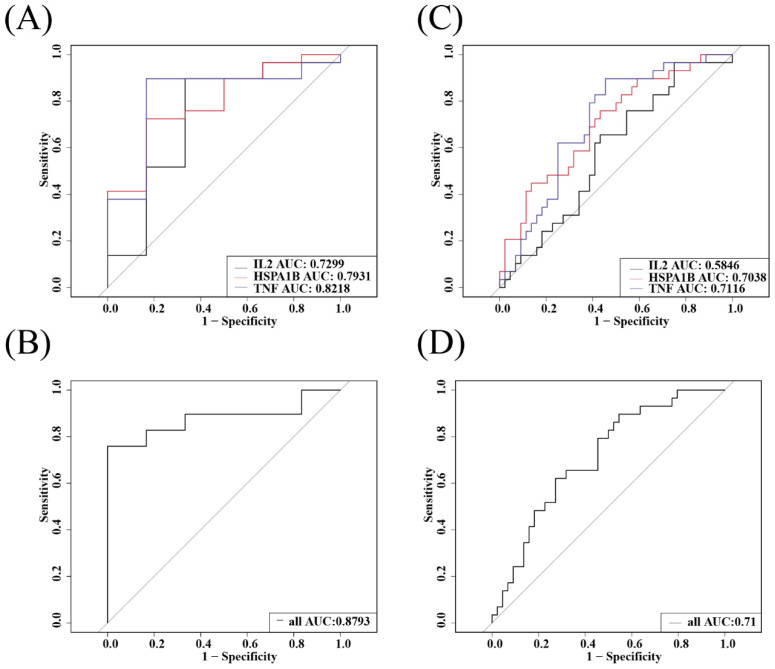
Joint analysis of IL-2 and hub genes. (**A**) ROCs showing the diagnostic performance of IL-2 and hub genes between *E.-coli*-infected patients and healthy individuals. (**B**) Combination of il-2 and hub genes between *E.-coli*-infected patients and healthy individuals. (**C**) The diagnostic performance of IL-2 and hub genes between *E. coli* infections and other bacterial infections. (**D**) Combination of il-2 and hub genes between *E. coli* infections and other bacterial infections.

**Figure 9:**
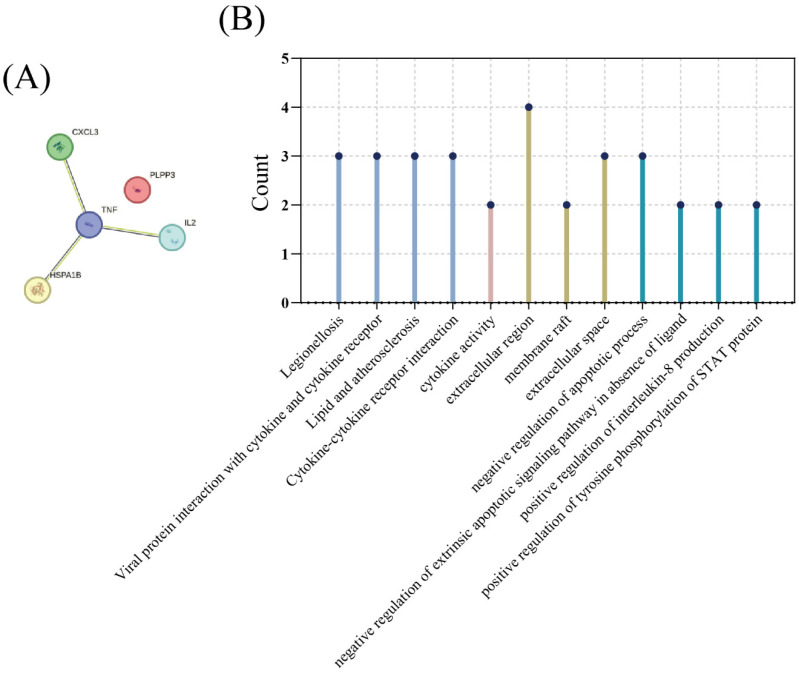
Functional enrichment analysis and PPI network diagram of CXCL3, HSPA1B, IL2, TNF, and PLPP3. (**A**) PPI network diagram. (**B**) BP, CC, MF, and KEGG analysis from left to right.

We conducted multicriteria ROC joint analysis of IL2, TNF, and HSPA1B. In differentiating healthy and *E. coli* infection groups, the AUC values of IL2 were 0.7299, HSPA1B was 0.7931, TNF was 0.8218, and the combination of the three was 0.8793. In differentiating *E. coli* infection from other bacterial infections, the AUC values of IL2 were 0.5846, HSPA1B was 0.7038, TNF was 0.7116, and the combined value of the three was 0.71 (Figure 8A–D). We found that, for the identification of *E. coli* infection and other infection groups, the addition of IL2 did not increase the AUC of the other two genes. However, IL2 increased the area under the curve of two other genes when distinguishing the healthy group from the *E.-coli*-infected group. Furthermore, we functionally analyzed all five genes using KEGG and GO and found that they were highly expressed in many pathways, such as cytokine–cytokine receptor interaction, membrane raft, the positive regulation of interleukin-8 production, and the positive regulation of tyrosine phosphorylation of STAT protein (Figure 9A,B).

## 4. Discussion

Sepsis is a life-threatening systemic inflammatory response mainly caused by a dysregulated response to pathogen infection, accompanied by severe inflammation and multiple-organ dysfunction [3,26]. As a global public health problem, sepsis can lead to higher mortality in critically ill patients [27]. Early diagnosis and timely and effective intervention are the keys to improving the prognosis and reducing the mortality of septic patients [28]. In addition, sepsis is the most common and serious disease among neonates. *Escherichia coli*, especially parenteral pathogenic *E. coli*, is the most common cause of death in neonatal sepsis due to its strong adaptability and strong pathogenic ability [29,30,31,32]. We retrospectively analyzed children with sepsis in our hospital, and the results showed that sepsis patients infected with *E. coli* accounted for 70.3% of all septic patients infected with Gram-negative bacteria. This reflects that *E. coli* is the main Gram-negative infectious bacteria in clinical practice. The high incidence of early-onset *Escherichia coli* sepsis suggests that we should pay attention to the immune features of *E. coli* infection, especially in neonates or young children [33]. Similarly, we also found that the age of the population infected with *E. coli* was significantly lower than that of the population infected with other bacteria. In summary, improving the understanding of the changes in the gene expression characteristics and clinical indicators of *E. coli* sepsis can help us understand the pathogenesis of infection and promote early diagnosis and treatment [12].

This study evaluated the difference between the cohorts with sepsis caused by *E. coli* infection and other bacterial infections and explored the key modules based on WGCNA. Based on DEGs, WGCNA, and MCODE, key genes associated with *E. coli* infection were discovered, including CXCL3, HSPA1B, TNF, and PLPP3. Then, we validated these signature genes in the external validation set and found that TNF and HSPA1B had greater values. At the same time, the correlation between immune cell infiltration and signature genes in septic patients infected with *E. coli* was analyzed using the CiberSort algorithm. In addition, we retrospectively collected data on clinical indicators from a total of 109 septic patients. The clinical index characteristics of patients infected with *E. coli* were observed and compared. We used the IL-2 proteins with significant differences in clinical indicators and the abovementioned key genes in the external validation set to analyze their correlation with *E. coli* infection.

IL-2 is a growth factor for T cells, and it plays a promotional role in T-cell growth and transformation [34]. IL-2 maximizes the toxic effects of T cells and increases the activity of natural killer cells. Lower levels of IL-2 were observed in the *E. coli* group in the present study, which may predict the downregulation of T-cell proliferation. In addition, IL-2 induced the gene expression of TNF-α in monocytes and macrophages [35], and TNF was mentioned in our study.

TNF is expressed on activated macrophages and lymphocytes, among other cell types, and it can induce cell survival or death of cell lines [36]. As a pleiotropic cytokine, TNF-α has strong proinflammatory and immunomodulatory properties [37]. Some studies have found that after continuous venous hemofiltration for severe sepsis, the secretion level of TNF-α was significantly reduced in patients [38]. In septic patients with bone marrow stromal cell (BMSC) transplantation failure, TNF-α was significantly upregulated [39]. Therefore, TNF is often associated with the inflammatory state of sepsis. However, we found higher expression in *E. coli*-infected sepsis, which may reflect a stronger inflammatory response and immune response. Our index analysis of clinical septic patients also showed that *E. coli* had a higher expression level of TNF-α, but no statistically significant difference was observed (Table 1).

Heat shock 70 kDa protein 1B (HSPA1B), also known as heat shock 70 kDa protein 2 (HSP70-2), belongs to the heat shock protein family [40]. It responds to organisms under proteotoxic stress by enhancing cell viability and promoting protein damage repair [41]. In addition, under cellular stress conditions, it is critical for maintaining protein homeostasis, such as assisting in protein folding [42]. Studies have found that HSPA1B-179C > T affects the production of HSP70 and is a key determinant of individual susceptibility to various inflammatory diseases [43]. In addition, HSP70 secreted into the extracellular environment can also lead to the upregulation of the expression of proinflammatory factors such as TNF by stimulating signal transduction cascade reactions [44]. This finding also suggests that HSPA1B may have a potential effect on TNF, the first signature gene we found.

In addition to analyzing the hub genes, we also analyzed the common indicators of clinical patients. One study found that the total bilirubin content of Gram-negative bacteria (22.5 μ mol/L) in infected patients was higher than that of Gram-positive bacteria (13.8 μ mol/L) [45]. This was also similar to our finding. We found that the total bilirubin and indirect bilirubin in patients with *E. coli* infection were significantly higher than those in patients with other bacterial infections (*p* = 0.033, 0.031). In addition, we performed immune cell infiltration analysis on the GSE6269 data and found that neutrophil counts in *E. coli* infection were significantly lower than those in other bacterial infections. This was also consistent with the result of our clinical retrospective analysis of data; that is, the proportion of neutrophils in patients with *E. coli* infectious sepsis was lower than that of patients with other bacterial infections. The depletion of peripheral B cells is associated with immunosuppression and immune system abnormalities during sepsis. Some studies have found that there is a high level of naive B cells in septic shock patients [46]. However, we found that naive B cells were high in *E.-coli*-infection-associated sepsis, which may be related to the depletion of peripheral blood B cells.

We analyzed the *E. coli* infectivity signature genes based on published data and combined this analysis with the clinical indicators commonly used in our hospital. For patients with sepsis caused by *E. coli* infection, early diagnosis and appropriate treatment are critical to improve clinical outcomes. By combining the information of signature genes and clinical indicators, we can establish a more accurate prediction model for the diagnosis of septic sepsis risk assessment and provide more individualized treatment plans.

Our research also faced specific constraints. The data included in this investigation were obtained from openly accessible databases and were derived from a limited sample size, potentially introducing selection bias. However, the validity of our investigation was verified using an independent validation database. Additionally, the number of patients with clinical sepsis was small, and the data were obtained only from a single center and, thus, have only reference value. Lastly, to confirm the study’s findings even further, more clinical samples and molecular tests are needed.

## Figures and Tables

**Table 1:** Nine candidate hub genes are shown in Figure 2A, and the genes were included in the MEgreen module and MCODE analysis of WGCNA. Four genes were obtained via WGCNA, and two genes were obtained after MCODE analysis.

Nine Genes in Figure 2A	WGCNA	MCODE
LOC728830	HSPA1B	HSPA1B
EDN1	PPAP2B	TNF
HSPA1B	CXCL2	
PPAP2B	TNF	
CXCL2		
TNF		
LOC643930		
LOC387763		
LOC642093		

**Table 2:** Common clinical indicators of patients with *E. coli* and other bacterial infections.

Characteristics	*E. coli* (*n* = 26)	G^+^ (*n* = 72)	*P E. coli* vs. G^+^	G^−^ (*n* = 11)	*P E. coli* vs. G^−^
Gender, F, *n* (%)	9 (34.6)	32 (40.5)	0.263	7 (58.3)	0.206
Age (month) median (IQR)	2 (0, 11)	24 (4, 72)	0.000	10.5 (2.3, 4.5)	0.3680
Laboratory parameters median (IQR)					
Albumin (g/L)	36.4 (33.1, 37.2)	39.5 (35.7, 43.3)	0.002	36.5 (33.8, 38.5)	0.370
Globulin (g/L)	18.3 (16.6, 20.5)	23.3 (17.4, 27.3)	0.004	20.0 (18.3, 23.6)	0.342
Total bilirubin (mol/L)	22.8 (8.7, 137.8)	8.6 (5.1, 14.6)	0.033	9.2 (5.5, 75.0)	0.071
Indirect bilirubin (mol/L)	14.1 (7.0, 122.9)	6.7 (3.8, 11.1)	0.031	7.3 (4.3, 28.5)	0.019
Adenosine deaminase (U/L)	10.1 (5.5, 13.6)	13.95 (9.5, 18.8)	0.033	13.7 (6.3, 22.4)	0.173
Prealbumin (g/L)	100.3 (61.2, 118.4)	139.1 (108.1, 188.6)	0.000	110.9 (91.3, 132.7)	0.063
Creatine kinase (U/L)	77.4 (50.0, 131.5)	123.5 (60.3, 271.8)	0.023	72.0 (59.0, 84.0)	0.447
Neutrophil (%)	46.8 (37.4, 61.5)	61.5 (41.8, 77.1)	0.037	61.0 (21.3, 90.4)	0.578
Monocyte (%)	9.1 (6.8, 11.9)	4.8 (6.7, 8.4)	0.006	5.6 (3.0, 8.6)	0.043
TNFα (ng/mL)	2.4 (1.9, 6.0)	2.1 (1.4, 4.1)	0.332	1.8 (1.6, 2.1)	0.619
IL2 (pg/mL)	2.1 (1.2, 3.1)	2.8 (2.4, 4.2)	0.023	2.8 (2.0, 4.85)	0.115

## Data Availability

The data that support the findings of this study are available from the corresponding author upon reasonable request. The data are not publicly available due to [The data contains patient privacy, is stored in the hospital database, and is not publicly available for a short period of time].
